# The Prevalence of Immunologic Injury in Renal Allograft Recipients with *De Novo* Proteinuria

**DOI:** 10.1371/journal.pone.0036654

**Published:** 2012-05-07

**Authors:** Qiquan Sun, Song Jiang, Xue Li, Xianghua Huang, Kenan Xie, Dongrui Cheng, Jinsong Chen, Shuming Ji, Jiqiu Wen, Mingchao Zhang, Caihong Zeng, Zhihong Liu

**Affiliations:** Research Institute of Nephrology, Jinling Hospital, Nanjing University School of Medicine, Nanjing, China; University of Colorado School of Medicine, United States of America

## Abstract

Post-transplant proteinuria is a common complication after renal transplantation; it is associated with reduced graft and recipient survival. However, the prevalence of histological causes has been reported with considerable variation. A clinico-pathological re-evaluation of post-transplant proteinuria is necessary, especially after dismissal of the term “chronic allograft nephropathy,” which had been considered to be an important cause of proteinuria. Moreover, urinary protein can promote interstitial inflammation in native kidney, whether this occurs in renal allograft remains unknown. Factors that affect the graft outcome in patients with proteinuria also remain unclear. Here we collected 98 cases of renal allograft recipients who developed proteinuria after transplant, histological features were characterized using Banff scoring system. Cox proportional hazard regression models were used for graft survival predictors. We found that transplant glomerulopathy was the leading (40.8%) cause of post-transplant proteinuria. Immunological causes, including transplant glomerulopathy, acute rejection, and chronic rejection accounted for the majority of all pathological causes of proteinuria. Nevertheless, almost all patients that developed proteinuria had immunological lesions in the graft, especially for interstitial inflammation. Intraglomerular C3 deposition was unexpectedly correlated with the severity of proteinuria. Moreover, the severity of interstitial inflammation was an independent risk factor for graft loss, while high level of hemoglobin was a protective factor for graft survival. This study revealed a predominance of immunological parameters in renal allografts with post-transplant proteinuria. These parameters not only correlate with the severity of proteinuria, but also with the outcome of the graft.

## Introduction

Post-transplant proteinuria is a common complication after renal transplantation. It is found in 25% of renal allograft recipients at 6 months [1], and nearly 50% at 1 year after transplantation [2]. The development of proteinuria is associated with reduced graft survival, independent of other risk factors, including glomerular pathology, graft function, and acute rejection [2], [3]. If urine protein is at the level of nephrotic syndrome, half of the patients will lose their graft within 2 years [4]. Even low-grade proteinuria is correlated with decreased graft survival [5], [6]. Nevertheless, proteinuria is also an independent risk factor for both cardiovascular and non-cardiovascular death [7], [8]. As a result, post-transplant proteinuria is becoming a significant barrier to both renal allograft and recipient survival.

The pathogenesis of proteinuria is complex. It can originate from both the native kidney and the allograft [9], [10], and may be caused by both glomerular damage and interstitial/tubular injury. Although this has been known for some time [11], [12], the overall clinico-histological features of patients with post-transplant proteinuria are far from clarified. The prevalence of histological causes reported by different centers has been quite different [1], [2], [4], [8], [12]. “Chronic allograft nephropathy,” which has been defunct as a term since 2005 [13] had also been counted as an important cause of proteinuria [2], [4], [9]. Urine protein can promote interstitial inflammation [14] in patients with kidney diseases, however, whether post-transplant proteinuria shares the same mechanism in inducing allograft injury need to be clarified. Moreover, factors that affect the graft outcome in patients with proteinuria also remain unclear. Thus, a clinico-pathological reevaluation of post-transplant proteinuria under the current Banff classification is necessary.

This study was performed to evaluate the overall clinical features and histological spectrum of post-transplant proteinuria. We unexpectedly revealed a high prevalence of immunological parameters in these patients, and moreover, these factors were correlated with the severity and outcome of the grafts. These findings question current strategies of managing post-transplant proteinuria.

## Materials and Methods

### Patients

Patients were selected from renal transplant recipients developing proteinuria from Jan. 2005 to Dec. 2008 at the Research Institute of Nephrology, Jinling Hospital, Nanjing University School of Medicine. Proteinuria is defined as urine protein over 0.4 g/d measured in 24-h collections by colorimetric methods. Inclusion criteria were as follows: (1) renal transplant recipients, (2) *de novo* proteinuria >0.4 g/d, (3) aged 18–60 years old, (4) having received baseline renal biopsies and index renal biopsy when proteinuria emerged, and (5) under follow-up for no less than 1 year. Patients who received sirolimus treatment were excluded because the incidence of proteinuria depends on the proportion of patients receiving this medicine. Patients in whom proteinuria emerged immediately after transplantation and declined over time were also excluded as this may have been related to the native kidney and have less influence on long-term graft survival.

Patients were followed at our institution, and all patients had a thorough evaluation once per week during the first 3 months, then once every 2 weeks until 6 months, monthly till the end of the first year, and bi-monthly thereafter. Data were recorded using a web-based recording system. Proteinuria was screened by urine test strips cassette with the URISYS 2400 urinalysis analyzer (Roche Diagnostics GmbH, Mannheim, Germany) at each visit, and by 24-h collection at every 6 months, or whenever the screening test was positive. The urine protein level was measured in 24-h collections by colorimetric methods. Urine n-acetyl-β-glucosaminidase (β-NAG) [15] and retinol binding protein (RBP) [16], [17] were used to evaluate the tubular injury. Graft function was evaluated with estimated glomerular filtration rate (eGFR).

### HLA-I, II antibody detection and lymphocytotoxic crossmatch

IgG anti-HLA class I and class II antibodies in serum samples were detected by flow cytometric analysis using the methods described by Pei et al [18]. Sera with >10% reactivity for HLA class I and/or II were considered positive for the presence of anti-HLA antibodies. HLA-I, II antibodies were monitored pre- and post-transplant, and whenever a renal biopsy was performed. Pre-transplant screening for donor-specific alloantibodies was also performed through complement-dependent lymphocytotoxicity methods using the National Institutes of Health technique with undiluted complement (without wash).

### Renal allograft pathology

Baseline biopsies were performed in all allografts to exclude any ongoing disease in the transplanted kidney. Diagnostic biopsies were performed whenever proteinuria emerged. Two needle biopsy cores were obtained from each renal allograft for morphological study, which were then divided into two parts: one for formalin fixation and one for quick-freezing. Hematoxylin and eosin, periodic acid Schiff, methenamine-silver, and Masson staining were routinely used on the formalin-fixed tissue. Fresh frozen tissue was analyzed by immunofluorescence microscopy using a conventional panel of antibodies against IgG, IgM, IgA, C3, C4, C1q, and C4d. Ultrastructural study were routinely studied using electron microscopy (EM) for all the samples. Since 2007, polyomavirus-associated nephropathy was routinely screened using the anti-SV-40 large T antigen antibody on all the biopsies. Histological features were scored using the latest Banff scoring criteria [13], [19], [20], [21] and CADI scoring [22]. All biopsies contained at least ten glomerular and two arterial sections. The pathology lesions are sorted into acute rejection, chronic rejection, TG, IF/TA, and de novo or recurrent glomerular diseases, such as IgA nephropathy, membranous nephropathy (MN), and focal segmental glomerulosclerosis (FSGS). The definition of the diagnosis terms were listed in table 1. Twenty protocol biopsy samples taken over one year post transplantation were randomized selected as controls.

**Table 1:** Definition of the diagnosis terms.

Term	Abbreviation	Definition
Transplant glomerulopathy	TG	Pathological featured duplication of the GBMs [21], and excluding other conditions that might result in similar histological changes, confirmed by EM.
Chronic rejection	CR	Including chronic active antibody-mediated rejection, chronic active T-cell mediated rejection [19], TG was excluded considering its high prevalence and unique histological feature.
Acute rejection	AR	Including acute antibody-mediated rejection, acute T-cell mediated rejection [19]
IgA nephropathy	IgAN	Dominant or codominant staining with IgA in glomeruli by immunofluorescence or immunoperoxidase [38], excluding TG.
Tubular atrophy and interstitial fibrosis	TA/IF	Interstitial fibrosis and tubular atrophy, with no evidence of any above specific etiology [19]

### Initial immunosuppression

Two primary immunosuppressive protocols were used in the course of this study: cyclosporine A (CsA), mycophenolate mofetil (MMF) and steroids or tacrolimus, MMF, and steroids. The main immunosuppressive protocols were CsA+MMF+steroid during June 2004 to June 2006, and tacrolimus+MMF+steroid since July 2006. AZA instead of MMF was also used before 2000. Induction therapy with daclizumab or basiliximab could also be used. The maintenance doses of tacrolimus and CsA were adjusted to target specific trough levels: 6–12 ng/mL during the first 6 months, 4–8 ng/mL thereafter for tacrolimus, and 150–250 ng/mL during the first 6 months and 100–200 ng/mL thereafter for CsA.

### Management of Proteinuria

Patients were given angiotensin-converting enzyme inhibitors and/or angiotensin receptor blockers when proteinuria occurred. For patients developing TG, the dose of MMF was increased to 1.5 g/d if the current dose was lower than 1.5 g/d. Additional immunoadsorption was used too if there were high levels of HLA class-I or II antibodies.

### Statistics

Descriptive statistical values are expressed as means±SD. Analyses were performed using the SPSS software (ver. 15.0; SPSS, Chicago, IL, USA). A *t*-test was used for comparing means and a chi-squared test was used for testing the significance of categorical variables. Survival curves were estimated using the Kaplan-Meier method, and differences were evaluated by the log-rank test. Cox proportional hazard regression models were used to adjust for the potential confounding effects of variables with statistical differences between the groups to evaluate the association between predictor variables and graft survival. A *p* value of <0.05 was considered to indicate statistical significance.

## Results

### Baseline Characteristics

Ninety-eight renal transplant recipients who developed proteinuria were included; there were 77 males and 21 females, all were negative for HLA-I and II antibodies before transplantation. The emerging time of proteinuria was 4.19±2.96 years (ranged from 1months to 10 years) post transplant, and the biopsies were performed immediately after protein emerging. As to the primary diseases that caused renal failure, 3 were diabetic nephropathy, 3 were FSGS, 4 were IgAN, 2 were membranous nephropathy, 1 were HSPN and 1 were crescent nephritis. Two were polycystic kidney disease. As the surveillance of kidney disease was not performed in China, the majority of patients were found to be at end stage renal failure with not native renal biopsies available. Baseline characteristics are listed in Table 2. The mean level of proteinuria was 2.2±2.0 g/d. A total of 34.7% of patients also showed different degrees of hematuria.

**Table 2:** Baseline characteristics of patients that developed post-transplant proteinuria.

Parameter	Value
*N*	98
Gender (male/female)	77 (78.6%)/21 (21.4%)
Age (years)	40.6±11.8/47.5±10.0
Onset of proteinuria (years post transplantation)	4.19±2.96
Peritoneal/hemodialysis before transplantation	3/95 (3.1%/96.9%)
Post-transplant complications	
Diabetes	10(10.2%)
Hepatitis	15(15.3%)
Pneumonia infection	14(14.3%)
Acute rejection	20(20.4%)

When the biopsies were performed, 41 patients used tacrolimus+MMF+steroid as immunosuppressive protocols and 46 patients were on CsA+MMF+steroid. There were no differences between the two groups on baseline characteristics such as age, gender, emerging time of proteinuria, etc. The degree of proteinuria were similar (2.11±1.76 vs 2.28±1.06 g/d, p = 0.700). The other eleven patients received CsA+Aza+steroid as immunosuppressive protocols. In addition, those patients were randomized matched with 20 recipients who received protocol biopsies 6 months after the transplant surgery. There were no differences in recipients' age, gender, baseline immunosuppressant, and post-transplant complications between the control group and patients with proteinuria.

### Characteristics of patients with different degrees of proteinuria

Compared with control group, post-transplant proteinuria was correlated with higher level of serum creatinine (2.86±2.36 vs 1.06±0.32 mg/dL, p<0.05), cholesterol (5.48±2.01 vs 3.72±0.72 mmol/L, p<0.05), triglyceride (1.56±0.68 vs 1.10±0.36, p<0.05), and lower level of serum albumin (34.6±5.4 vs 41.8±4.6, p<0.05).

**Figure 1:**
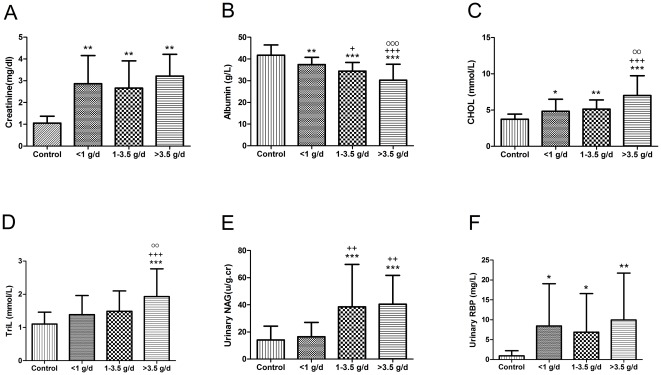
Characteristics of patients with different ranges of proteinuria. *, P<0.05 compared with control group; **, P<0.01 compared with control group; ***, P<0.001 compared with control group. ^+^, P<0.05 compared with urinary protein<1 g/d group; ^++^, P<0.01 compared with urinary protein<1 g/d group; ^+++^, P<0.001 compared with urinary protein<1 g/d group. ^O^, P<0.05 compared with urinary protein 1–3.5 g/d group; ^OO^, P<0.01 compared with urinary protein 1–3.5 g/d group; ^OOO^, P<0.001 compared with urinary protein 1–3.5 g/d group.

In native kidney diseases, >3.5 g/d usually is defined as nephritic range proteinuria, while <1 g/d usually is tubulointerstitial non-neprotic proteinuria. We divide the patients into 3 groups based on the level of urine protein: urine protein >3.5 g/d, 1–3.5 g/d, and <1 g/d. There was no difference between groups in patient age or gender. The onset time was significantly later in patients with high levels (>3.5 g/d) of proteinuria compared with patients with low levels (<1.0 g/d) of proteinuria (3.45±2.85 vs 5.80±3.01 years, p<0.05). Higher levels of proteinuria were correlated with lower serum albumin, higher levels of blood lipid, and urinary NAG (u/g.cr; Figure 1).

**Figure 2:**
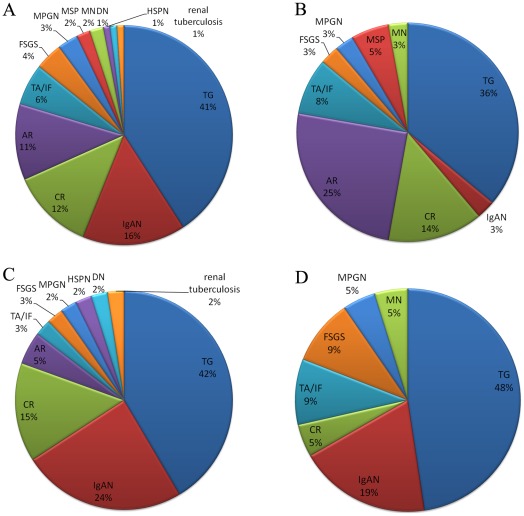
Histological causes of post-transplant proteinuria. Figures shows histological causes of proteinuria in all patients (1A), urine protein <1 g/d (1B), between 1–3.5 g/d (1C), and over 3.5 g/d (1D). TG was the leading cause of overall post-transplant proteinuria and each subgroup, followed by IgAN, chronic rejection (TG excluded), and acute rejection. For proteinuria below 1.0 g/d, acute rejection was the second most important cause of proteinuria, accounting for 25% in this subgroup; only one (3%) patient was diagnosed as having IgAN. For proteinuria between 1–3.5 g/d, the incidence of acute rejection decreased to 4.9%, while the incidence of IgAN increased to 24.4%. In proteinuria over 3.5 g/d, IgAN accounted for 19%, and no patient was diagnosed as having acute rejection. TG, transplant glomerulopathy; IgAN, IgA nephropathy; AR, acute rejection; CR, chronic rejection; TA/IF, tubular atrophy and interstitial fibrosis; FSGS, Focal segmental glomerulosclerosis; MPGN, Membranoproliferative glomerulonephritis; HSPN, Henoch-Schönlein purpura nephritis; DN, diabetic nephropathy; MSP, mesangial proliferative glomerulonephritis; MN, Membranous nephropathy.

TG was the leading cause of overall post-transplant proteinuria; it accounted for 41% of proteinuria in all ranges, followed by IgAN for 16%, chronic rejection (TG excluded) for 12%, and acute rejection for 11% (Figure 2A).

For proteinuria below 1.0 g/d, TG was still the leading cause. While acute rejection was the second most important cause of proteinuria, it accounted for 25% of this subgroup, followed by chronic rejection (14%). Only one (3%) patient in this group was diagnosed as having IgAN (Figure 2B). For proteinuria between 1–3.5 g/d, TG was also the most important lesion in histology; however, the incidence of acute rejection decreased to 4.9%, while the incidence of IgAN increased to 24.4% (Figure 2C). In the group of proteinuria over 3.5 g/d, TG accounted for 47.6% of all causes, and IgAN accounted for 19%, while no patient was diagnosed as having acute rejection (Figure 2D).

### Characteristics of patients with different pathologies

We compared the clinical characteristics of six major causes of proteinuria (Table 3). TG was the latest lesion that could cause proteinuria, with a diagnosis time of 5.21±2.71 years post-transplant, followed by IgAN and chronic rejection. Acute rejection was the earliest lesion, occurring at 1.20±1.31 years post-transplant, which was significantly earlier than TG (p<0.05).

**Table 3:** Characteristics of patients with different histologies.

	TG	IgAN	CR	AR	TA/IF	FSGS
	(*n* = 40)	(*n* = 15)	(*n* = 12)	(*n* = 11)	(*n* = 6)	(*n* = 4)
***Basic Characteristics***						
Age (years)	44.7±12.3	40.9±8.1	37.8±1.8	36.4±11.6	44.3±12.6	36.8±7.9
Gender (male%)	31 (77.5%)	13 (86.7%)	8 (66.7%)	8 (72.7%)	5 (83.3%)	3 (75.0%)
Diagnosis time (years post Tx)	5.21±2.71	4.61±2.82	4.56±3.30	1.20±1.31^a^	3.99±3.66	2.58±2.10
***Urine examination***						
Upro (g/24 h)	2.30±1.94	2.42±1.27	1.73±2.09	1.06±0.91^b^	2.98±3.52	3.25±2.34
<1 g/24 h	13 (32.5%)	1 (6.7%)^c^	5 (41.7%)	9 (81.8%)^d^	3 (50.0%)	1 (25.0%)
1–3.5 g/24 h	17 (42.5%)	10 (66.7%)	6 (50.0%)	2 (18.2%)^b^	2 (33.3%)	1 (25.0%)
>3.5 g/24 h	10 (25%)	4 (26.7%)	1 (8.3%)	0 (0)^e^	1 (16.7%)	2 (50.0%)
Hematuria	17 (42.5%)	8 (53.3%)	1 (8.3%)	3 (27.3%)	0 (0)^f^	0 (0)
NAG (u/g.cr)	29.5±20.7	24.8±11.2	25.9±7.0	35.5±36.9	22.0±18.3	33.5±25.3
RBP (mg/L)	6.03±8.28	1.6±2.2	18.2±13.1^g^	7.4±9.4	13.0±16.0	6.9±8.9
***Blood examination***						
Albumin (g/L)	33.4±5.6	35.0±4.9^#^	36.3±4.8	38.0±2.5^#^	34.8±5.4	37.0±4.1
≥35 g/L	18 (45%)	8 (53.3%)	8 (66.7%)	10 (90.9%)^h^	2 (33.3%)	3 (75.0%)
<35 g/L	22 (55%)	7 (46.7%)	4 (33.3%)	1 (9.1%)^h^	4 (66.7%)	1 (25.0%)
Scr (mg/dL)	2.69±1.67	2.14±2.82^b^	2.44±1.16	4.21±2.59	4.29±5.10	4.50±3.29
<1.24 mg/dL	3 (7.5%)	7 (46.7%)^i^	0 (0)	0 (0)	1 (12.7%)	0 (0)
≥1.24 mg/dL	37 (92.5%)	8 (53.3%)	12 (100%)	11 (100%)	5 (83.3%)	4 (100%)
CHOL (mmol/L)	5.37±1.66	5.40±1.40	5.39±1.38	4.88±2.50	5.94±2.27	5.96±1.10
TriG (mmol/L)	1.58±0.72	1.40±0.45	1.50±0.75	1.35±0.46	1.48±0.28	2.20±0.95^j^
HLA-I antibodies	12.6±17.6	3.93±1.83	2.49±2.08	18.2±26.5	0.7	1.2
HLA II antibodies	23.62±27.91	4.29±3.47	2.44±1.21	7.76±9.64	1.3	0.3
Hemoglobin	9.67±2.44	11.67±2.31^k^	10.19±2.22	9.48±2.55	10.33±3.31	11.0±4.06
>12 g/L	6 (15.0%)	8 (53.3%)	3 (25.0%)	1 (9.1%)	1 (16.7%)	1 (25.0%)
9–12 g/L	16 (40.0%)	5 (33.3%)	7 (58.3%)	5 (45.5%)	2 (33.3%)	2 (50.0%)
6–9 g/L	15 (37.5%)	2 (13.3%)	2 (16.7%)	4 (36.4%)	3 (50%)	1 (25.0%)
<6 g/L	3 (8.1%)	0 (0)	0 (0)	1 (9.1%)	0 (0)	0 (0)

TG: transplant glomerulopathy; IgAN: IgA nephropathy; CR, chronic rejection; AR, acute rejection; TA/IF: tubular atrophy and interstitial fibrosis; Tx, transplantation; Upro, urine protein; NAG, n-Acetyl-β-glucosaminidase; RBP, retinol binding protein; SCr, serum creatinine; CHOL, cholesterol; TriL, Triglyceride.

a , P<0.01, AR vs. TG, IgAN, CR;

b , P<0.05, IgAN vs. AR group;

c , p<0.05, IgAN vs. AR, CR, and TA/IF;

d , P<0.05, AR vs. IgAN, FSGS and TG;

e , p<0.05, AR vs. FSGS;

f , P<0.05, TA/IF vs. TG and IgAN;

g , P<0.05, CR vs. TG, IgAN;

h , P<0.05, AR vs. TG, IgAN, TA/IF;

i , P<0.05, IgAN vs. TG, CR, AR;

j , P<0.05, IgAN vs. FSGS, AR;

k , P<0.05, IgAN vs. TG, AR.

Patients in the TG group had a higher level of de novo HLA class I and class II antibodies, especially for class II antibodies. More than 40% patients with TG were positive for HLA class II antibodies (over 10% in PRA). The acute rejection group had a higher level of HLA class I antibodies, but the difference was not statistically significant. Hematuria could be found in TG, IgAN, and acute rejection, but seldom in chronic rejection, TA/IF, or FSGS groups.

TG could cause different ranges of proteinuria, whereas in IgAN group, most patients (93.4%) had proteinuria over 1 g/d, compared with 18.2% (p<0.05) in the acute rejection group. Half of the patients in the FSGS group had proteinuria over 3.5 g/d.

With respect to graft function, all patients in the acute rejection and chronic rejection groups, and 92.5% in the TG group had impaired graft function, while nearly half of the patients in the IgAN group had normal graft function. Post-transplant proteinuria in different groups correlated with different incidences of hypoproteinemia and hyperlipidemia. Compared with the acute rejection group, the TG and IgAN groups had significantly higher incidences of hypoproteinemia (p<0.05). The acute rejection group had the lowest serum lipid levels among the six groups.

### Prevalence of immunological parameters in histology

**Figure 3:**
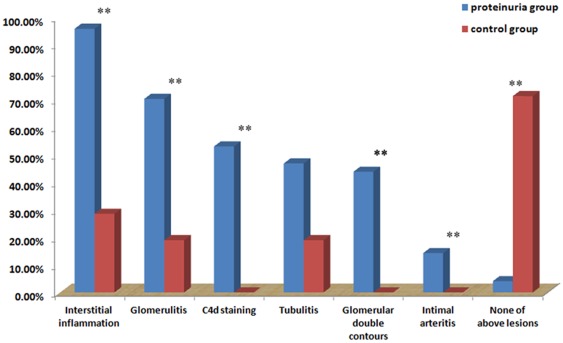
Prevalence of histological lesions related to immune activity. Most (95.9%) of the patients with proteinuria had at least one kind of immunological lesion, based on histology. Interstitial inflammation was the most common lesion, which could be detected in 95.9% patients with proteinuria, followed by glomerulitis in 70.4%, C4d deposition in peritubular capillaries in 53.1%, tubulitis in 46.9%, and intimal arteritis in 14.3%. While in the control group, only few recipients have immunological lesion. *, p<0.05 comparing with control group; **, p<0.001 comparing with control group.

Regardless of histological diagnosis, we found that almost all patients (95.9%) with proteinuria suffered from one or more kinds of immunological lesions, as detected by histology. Among those lesions, interstitial inflammation was the most common and could be found in 95.9% patients with proteinuria, followed by glomerulitis in 70.4%, C4d deposition in peritubular capillaries in 53.1%, tubulitis in 46.9%, and intimal arteritis in 14.3%. As a typical lesion of TG, glomerular double contours could be detected in 43.9% patients. Only four patients (4.1%) were negative for all immunological lesions. The incidences of above lesions were significantly higher than the matched group of graft receiving protocol biopsies (Figure 3). Looking into the causes of proteinuria, there was high prevalence of interstitial inflammation in each group. TG group has a higher incidence of tubular atrophy than acute rejection group and a higher incidence of C4d deposition comparing with IgAN (table 4).

**Table 4:** Histologic findings of patients with different histologies.

	TG	IgAN	CR	AR	TA/IF	FSGS
	(*n* = 40)	(*n* = 15)	(*n* = 12)	(*n* = 11)	(*n* = 6)	(*n* = 4)
Interstitial inflammation, n (%)	37 (92.5%)	12 (80.0%)	11 (91.7%)	11 (100.0%)	5 (83.3%)	3 (75.0%)
Glomerulitis, n (%)	33 (82.5%)	11 (73.3%)	9 (75.0%)	9 (81.8%)	2 (33.3%)	3 (75.0%)
Tubulitis, n (%)	18 (45.0%)	6 (40.0%)	7 (58.3%)	10 (90.9%)	2 (33.3%)	2 (50.0%)
Tubular atrophy, n (%)	36 (90.0%)*	14 (93.3%)	10 (83.3%)	5 (45.5%)	5 (83.3%)	3 (75.0%)
Interstitial infiltrate, n (%)	37 (92.5%)	12 (80.0%)	11 (91.7%)	11 (100%)	5 (83.3%)	3 (75.0%)
Plasma cell infiltrate, n (%)	19 (47.5%)	4 (26.7%)	7 (58.3%)	5 (45.5%)	2 (33.3%)	0 (0)
Interstitial fibrosis, n (%)	33 (82.5%)	9 (60.0%)	12 (100.0%)	7 (63.6%)	4 (66.7%)	2 (50.0%)
C4d deposition	28 (70.0%)#	4 (26.7%)	8 (66.7%)	7(63.6%)	1 (16.7%)	1 (25.0%)
Diffuse	22 (55.0%)#	2 (13.3%)	5 (41.7%)	3 (27.3%)	0 (0)	0 (0)
Focal	6 (15.0%)	2 (13.3%)	3 (25.0%)	4(36.4%)	1 (16.7%)	1 (25.0%)
Negative	12 (30.0%)#	11 (73.3%)	4 (33.3%)	4 (36.4%)	5 (83.3%)	3 (75.0%)
Intraglomerular C3 deposition, n (%)	22 (55.0%)*	13 (86.7%)	5 (41.7%)	1 (9.1%)	3 (50.0%)	2 (50.0%)
Diffuse	7 (17.5%)# *	10 (66.7%)	1 (8.3%)	1 (9.1%)	2 (33.3%)	2 (50.0%)
Focal	15 (37.5%)*	3 (20.0%)	4 (33.3%)	0 (0)	1 (16.7%)	0 (0)
Negative	18 (45.0%)*	2 (13.3%)	7 (58.3%)	10 (90.9%)	3 (50.0%)	2 (50.0%)
Intimal arteritis, n (%)	3 (7.5%)	0 (0)	1 (8.3%)	2(18.2%)	0 (0)	1 (25.0%)
small vessel fibrinoid necrosis	3 (7.5%)	1 (6.7%)	1 (8.3%)	1 (9.1%)	0 (0)	0 (0)
Small vessel thrombi	3 (7.5%)	1 (6.7%)	0 (0)	1 (9.1%)	0 (0)	0 (0)

* , p<0.05 vs AR group;

# , p<0.05 vs IgAN group.

### Factors correlated with the grade of urine protein

We also attempted to study the grade of urine protein using clinical and histological features. We found that the quantity of urine protein was strongly correlated with the incidence of intraglomerular C3 deposition (r = 0.293, p = 0.007), degree of tubular atrophy (Ct, r = 0.289, p = 0.008), glomerular sclerosis (Cg, r = 0.238, p = 0.009), and interstitial fibrosis (Ci, r = 0.227, p = 0.038). In contrast, it did not correlate with patient age, gender, degree of interstitial inflammation, C4d deposition, or graft function at diagnosis.

### Prognostic factors for graft survival

**Figure 4:**
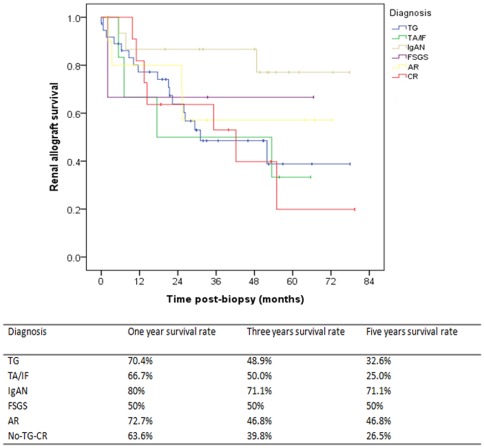
Graft survival for patients with different histological causes of proteinuria. IgAN has the best graft outcome, with a 5-year graft survival of 71.1%, while graft survival was very poor in patients with TG, TA/IF, and CR. TG, transplant glomerulopathy; IgAN, IgA nephropathy; AR, acute rejection; CR, chronic rejection; TA/IF, tubular atrophy and interstitial fibrosis; FSGS, Focal segmental glomerulosclerosis.

TG had a poor graft survival at 5-years post-diagnosis with 32.6%, similar to the outcome of IF/TA and non-TG chronic rejection. The 5-year graft survival in the acute rejection group was 46.8%. IgAN had the best 5-year graft survival of 71.1% in this cohort (Figure 4).

**Figure 5:**
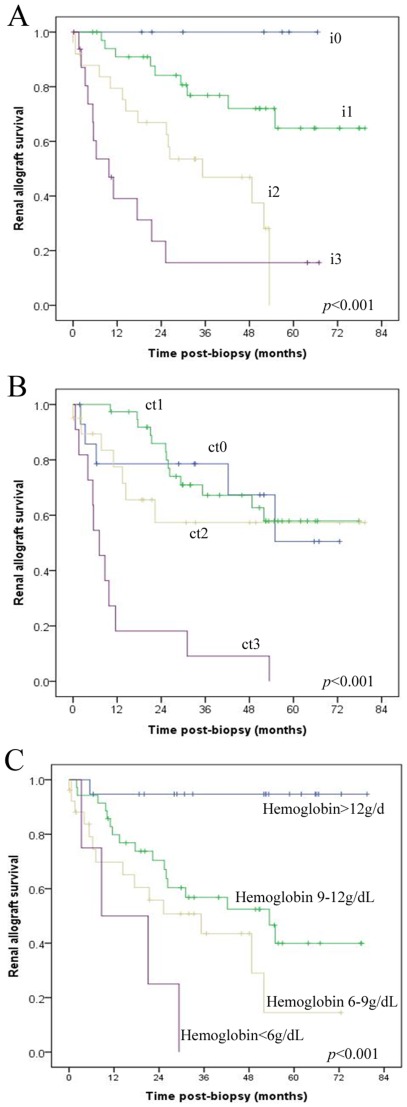
Risk factors correlated with the graft survival in patients with post-transplant proteinuria. Degrees of interstitial inflammation (A) and renal tubular atrophy (B) and levels of hemoglobin (C) were strongly correlated with graft survival. i 0–3, mononuclear cell interstitial inflammation grade 0–3; ct 0–3, tubular atrophy grade 0–3. [21]

We performed a Cox regression analysis, which included clinical factors such as age, gender, onset time of proteinuria, hematuria, NAG, serum creatinine, albumin, as well the diagnosis. Univariable analyses revealed that eGFR, range of proteinuria, degree of interstitial inflammation, score of vascular lesions, and degree of tubular atrophy were all risk factors for early graft loss. In contrast, level of serum hemoglobin and albumin, the diagnosis of IgAN were associated with graft survival. However, multivariate analyses revealed that only the degrees of interstitial inflammation (Figure 5A) and tubular atrophy (Figure 5B) were independent risk factors of graft loss, while levels of serum hemoglobin (Figure 5C) were independent protection factors for graft survival (Table 5).

**Table 5:** Independent risk factors and protective factors for transplantation kidney survival.

Variable	Univariate			Multivariate		
	HR	CI	P	HR	CI	P
Interstitial inflammation	2.772	1.859–4.134	<0.001	2.397	1.535–3.742	<0.001
Tubular atrophy	2.173	1.488–3.173	<0.001	2.270	1.546–3.334	<0.001
Serum hemoglobin	0.965	0.951–0.979	<0.001	0.971	0.953–0.990	0.003
Serum albumin	0.932	0.874–0.993	0.028			
eGFR	0.953	0.934–0.972	<0.001			
Urine protein	1.255	1.072–1.470	0.005			
Intimal arteritis	1.443	1.025–2.033	0.036			
IgA Nephropathy	0.294	0.090–0.961	0.043			

Although C4d deposition could be detected in 52 (53.1%) patients (39 diffuse, 13 focal), we did not find correlation between C4d deposition and graft outcome. Although TG group seemed to be correlated with a poor graft survival, univariate analyses didn't show TG is independent risk factor for graft loss.

## Discussion

This study outlined clinicopathological features of *de novo* post-transplant proteinuria under modern immunosuppressive protocols and the current Banff classification system. We revealed a predominance of immunological parameters in patients developing *de novo* proteinuria after transplantation. Immunological causes, including TG, acute rejection, and chronic rejection, accounted for 64.3% of all causes; indeed, almost all patients who developed proteinuria had histological immunological lesions in the graft, especially interstitial inflammation. Moreover, intraglomerular C3 deposition correlated with the grade of proteinuria, and the severity of interstitial inflammation was an independent risk factor for graft loss. These data suggest the involvement of immunological factors in graft dysfunction.

Immunological causes, including TG, acute rejection, and chronic rejection, account for the majority of post-transplant proteinuria. Among them, TG was the leading cause, accounting for 41% of proteinuria, covering different ranges of urine protein, and 42.5% of them were accompanied by hematuria. TG is regarded as a form of late antibody-mediated rejection, however, as it has featured glomerular damage differing to chronic rejection, we list it separately in this study. As reported [23], the TG group had a higher level of HLA class I and class II antibodies, especially class II antibodies. Patients with proteinuria caused by TG had a poor outcome, with only 32.6% 5-year graft survival. The high incidence and poor outcome of TG might account for the poor graft outcome of post-transplant proteinuria. It is noteworthy that the onset of proteinuria was at 5 years after transplantation in this group. This may explain why a recent study based on biopsies performed at 1-year post transplantation showed only a low incidence of TG [2]. However, TG was also the leading cause of graft loss in that group.

Another immunological entity, acute rejection, is an important cause of early post-transplant proteinuria and is related to poor graft survival. Proteinuria caused by acute rejection is characterized by early (within 1 year) occurrence and low levels (<1 g/d). It was rarely accompanied by hypoalbuminemia and lower levels of serum CHOL. This is likely due to the acute tubular damage caused by rejection, because a raised NAG level had been observed in this group. Although proteinuria caused by acute rejection is not as heavy as IgAN, the outcome is much worse. Our study was consistent with data reported by Halimi *et al.* [24], in which the incidence of early (within 3 months after transplantation) lower grade proteinuria and graft outcomes correlated with episodes of acute rejection. Unfortunately, they did not perform surveillance biopsies in their study, so it is unclear whether there were other coexisting lesions in those grafts.

Our data reveal a high prevalence of immunological lesions in the histology of renal allografts with *de novo* proteinuria. Regardless of histological cause, almost all patients who developed proteinuria had interstitial inflammation in the graft, which is much higher than in the grafts without urine protein. Although it is difficult to clarify the cause and consequence of inflammation, it is possible that urinary protein itself could stimulate interstitial inflammation. In native kidney, it is well known that protein overload can stimulate proximal tubular cells to synthesize chemokines, which may contribute to the chronic tubulointerstitial inflammation [14]. Our data suggest that this pathway maybe also exist in renal allograft recipients, even in the era of modern immunosuppresants. Anyway, this hypothesis needs to be further proved.

Never the less, interstitial inflammation was strongly correlated with graft outcome. Our data reveal that many factors correlated with graft outcome, including graft function (measured with eGFR), grade of proteinuria, scores of interstitial inflammation, vascular lesions and tubular atrophy, level of serum hemoglobin, and albumin. However, only degrees of interstitial inflammation (Figure 5A) and tubular atrophy (Figure 5B) were independent risk factors of graft loss. In contrast, levels of serum hemoglobin (Figure 5C) were independent protective factors for graft survival. Among risk factors, interstitial inflammation had the highest hazard ratio. Although a high prevalence of C4d-deposition had been noticed in this cohort, we didn't find correlation between C4d deposition and graft outcome, which is consistent with data from Sis et al based on a population of TG [25].

For the first time, we report that hemoglobin is an independent protective factor for renal allograft survival in patients who develop *de novo* post-transplant proteinuria. Specifically, anemia is an independent risk factor for graft loss. The estimated graft survival was 44.5 months for patients without anemia, while it was only 22.1 months for patients with hemoglobin between 6–9 g/dL and 13.4 months for patients with hemoglobin levels below 6 g/dL (Figure 5C). This is another important finding that may impact clinical practice, because anemia is common in renal allograft recipients with proteinuria and usually is believed to be related with eGFR. This finding is consisted with data from a multicenter clinical trial that showed complete correction of anemia reduce the rate of progression of chronic allograft nephropathy. [26]

Evidence that the degree of interstitial inflammation and anemia correlates with graft survival brings into question the traditional management of proteinuria, which consists primarily of angiotensin-converting enzyme inhibitors and/or angiotensin receptor blockers as the first-line treatment. [27], [28], [29], [30], [31], [32] Physicians may need to consider dealing with the underlying immunological activity and maintaining the hemoglobin at a higher level. A renal biopsy will still be necessary to assess the underlying histological causes to exclude non-immunological lesions, which may be more suitable for traditional management. Nevertheless, our data support the hypothesis that proteinuria-induced graft loss occurs via complex mechanisms other than the presence of glomerular disease [28].

Our study also revealed that another immunological lesion, intraglomerular C3 deposition, correlated with the grade of proteinuria. Cox regression analysis revealed that in addition to histological causes and onset time, proteinuria levels also correlated with intraglomerular C3 deposition, degree of allograft glomerulopathy, tubular atrophy, and interstitial fibrosis. However, it did not correlate with patient age, gender, degree of interstitial inflammation (i0–3), C4d deposition, or graft function at diagnosis. For the first time, we report that the degree of *de novo* post-transplant proteinuria correlated with the incidence of C3 deposition. C3 deposition in glomerulus is not rare in patients undergoing kidney diseases, and can even be detected in living donors during nephrectomy [33]. The significance of C3 deposition in the development of proteinuria remains to be clarified, although it has been reported that co-deposition of C3 with C4d is correlated with early graft loss [34]. We did not find a correlation between C3 deposition and graft survival in this cohort.

Because IgAN is the most common primary glomerulonephritis worldwide [35] and the recurrent rate is high after transplantation [36], [37], it is not surprising that renal allograft IgAN was common in this cohort. Our data suggest that IgAN is one of the main causes of proteinuria over 1 g/d, and is a relatively benign lesion, which had the best 5-year graft survival in the current cohort. Univariation analysis also showed the diagnosis of IgAN is protecting factor for graft loss. IgAN in this group should be regarded as *de novo* or recurrent, because baseline biopsies had been performed to exclude disease derived from the donor. The high incidence of IgAN in this group might be partly due to its high prevalence in the general population [35]. Although the 5-year graft looks good in this group, additional attention should be paid to these recipients because longer term observation revealed decreased graft survival [36].

Moreover, our study revealed that different grades of proteinuria emerging at different times correlated with different histological findings, which could explain why a different prevalence of histological causes had been previously reported from different centers [1], [2], [4], [8], [12]. Low-grade (<1 g/d) proteinuria was mostly associated with interstitial and tubular damage, such as acute rejection, chronic rejection, and TA/IF. Glomerular damage is likely involved in the development of high-grade proteinuria. Our data support the hypothesis that early proteinuria is usually correlated with acute allograft damage, such as acute rejection. Based on these data, β-NAG and RBP are helpful factors in distinguishing acute and chronic lesions. Acute lesions typically manifest as higher levels of NAG and lower levels of RBP. In contrast, chronic lesions are correlated with lower NAG and higher RBP. Due to the exclusion of proteinuria emerging immediately after transplantation, no acute tubular necrosis cases were observed in this group. Overall, based on the above data, it is not surprising that studies focused on proteinuria occurring at different times and/or of different ranges would lead to different histological findings. This is why rare TG was reported in patients developing proteinuria 1-year after transplantation, [2] while rare acute rejection was reported in patients with post-transplant nephrotic syndrome. [4]

It is necessary to acknowledge the lack of native renal biopsies in most recipients prior to transplantation, which prevented us from making a diagnosis of recurrent or *de novo* renal disease. However, in this group, excluding IgAN, only 13.3% are likely to have *de novo* or recurrent glomerulonephritis. The conclusion we draw is not limited, because we did not focus on patients with *de novo* or recurrent renal disease.

Overall, our study revealed a predominance of immunological parameters in renal allografts with post-transplant proteinuria. These parameters not only correlate with the severity of proteinuria, but also with the outcome of the graft. These findings are important because they bring into question current strategies of managing post-transplant proteinuria. Specifically, patients might benefit from the introduction of anti-inflammatory treatments.
